# An Exploration of the Impacts of the 2019 Floods in Townsville, Australia on Community Pharmacy Operations

**DOI:** 10.1017/S1049023X25101301

**Published:** 2025-08

**Authors:** Judith Singleton, Elizabeth McCourt, Kaitlyn Watson, Alexander Letts

**Affiliations:** 1. Faculty of Health, School of Clinical Sciences, Queensland University of Technology, Brisbane, Australia; 2. EARTH Research Group, QUT Resilience Center, Queensland University of Technology, Brisbane, Australia; 3. Royal Brisbane and Women’s Hospital, Metro North Health and Hospital Service, Brisbane, Australia; 4. Faculty of Pharmacy and Pharmaceutical Sciences, University of Alberta, Edmonton, Canada

**Keywords:** community pharmacy services, disaster, disaster relief planning, medication management, natural disasters

## Abstract

Between January 29 and February 11, 2019, the Townsville region in Australia experienced a major flooding event. This study explored impacts on affected community pharmacies. Semi-structured phone interviews were conducted with six pharmacists who worked in affected Townsville community pharmacies during this flood. De-identified transcript data were analyzed using reflexive thematic analysis. The thematic analysis yielded six themes – “financial impact on pharmacy owners,” “engagement with Local Disaster Coordination Center (LDCC) important,” “workload pressures,” “preparedness,” “medication supply impacts,” and “communication and collaboration.” Financial impacts to owners included loss of property (two pharmacies were completely flooded), purchase or hire costs of generators when power was lost, and loss of revenue from complete or early closure of pharmacies and when patients could not pay or did not have a prescription and did not return to the pharmacy after the event. Engagement with the LDCC assisted pharmacy responsiveness. Medication supply issues were experienced by patients whose houses had flooded, or who had left their prescriptions with pharmacies that had flooded. Opioid Replacement Therapy (ORT) program patients were also impacted due to communication difficulties between them, their clinics, and their pharmacies. Increased customer numbers by those whose regular pharmacy was closed, reduced staff numbers, and austere working conditions increased workload pressures. Pharmacists collaborated to consolidate resources with those whose pharmacy had closed, working in pharmacies that were open. This research highlights a critical need for improved flood preparedness among Townsville pharmacists. Regardless, they collaborated to ensure there were minimal critical medication delays.

## Event Description:

- **Event Type:** Flood
- **Event Onset Date:** Between January 29 and February 11, 2019
- **Location of Event:** Townsville, Queensland, Australia
- **Geographic Coordinates:**
- Latitude: 19°15′24.98″ South
- Longitude: 146°49′26.23″ East
- Elevation: 4m
- **Data Collected:** August 2019
- **Response Type:** Community Pharmacy Response

## Introduction

In late January 2019, an active monsoon trough and a slow-moving, low-pressure system over tropical northern Queensland caused consecutive days of consistent extremely heavy rainfall.^1^ Between January 29 and February 11, 2019, the Townsville region recorded record-breaking rainfall levels (more than 2000mm)^1^ which exceeded the region’s average annual rainfall.^2^ The Ross River Dam received a rainfall amount 3.8-times its capacity.^1^ Consequently, the Townsville region experienced major flooding which exceeded previous flood records over the previous 120 years.^2,3^ An estimated 3,300 private and commercial properties were damaged.^4^ Natural disasters, such as Queensland’s monsoon trough weather event, impact the community’s ability to access health care, including medications.^5^ Therefore, the aim of this study was to explore the impacts of this flood event on community pharmacy operations in Townsville, Queensland.

## Sources

This qualitative research study utilized semi-structured interviews which were conducted in August 2019. The interview questions (Supplementary Material; available online only) explored pharmacists’ experiences of the flood event utilizing questions relating predominantly to the Response phase of the Prevention-Preparedness-Response-Recovery (PPRR) disaster management model.^6^ The interview questions were modified from similar questions employed in two previously published studies of bush fire events.^7,8^ Prior to data collection, ethical approval was obtained from the Queensland University of Technology (Brisbane, Australia) Human Research Ethics Committee (Approval Number 1900000230).

### Sampling Strategy and Participant Recruitment

Ten pharmacies were confirmed as flooded or cut off by floodwaters, with seven owners/managers agreeing to their staff being interviewed. Eligibility criteria for participants were that they had to be a registered pharmacist with the Australian Health Practitioner Agency (AHPRA; Brisbane, Australia) and had to be working in Townsville between January 29, 2019 – February 11, 2019 in community pharmacies which were affected in some way by flooding. Pharmacists who were willing to participate then signed an informed consent document and an interview time was arranged. Participants were also asked if they knew of any other pharmacists who met the eligibility criteria and might be willing to participate in the research. A total of six pharmacists were interviewed.

### Data Collection and Analysis

Authors KW and JS conducted the interviews over the phone, and only the interviewee and either KW or JS were present during the interviews. Both KW and JS are registered pharmacists with AHPRA but were not known to any of the interviewees. Being pharmacists, KW and JS could appreciate nuanced responses and probe further, where relevant. Each interview was recorded digitally and averaged 20 minutes. To ensure confidentiality, prior to their interview, each participant was assigned a unique identification code which was used in the audio recording. The audio files were then transcribed using *intelligent verbatim* by AL. Since participants’ responses often addressed more than one question at a time, the interviews were analyzed in their entirety rather than analyzing each question’s data separately. Whilst a predominantly deductive approach was taken mapping back to the PPRR framework, a formal deductive approach, such as grounded theory (as per Corbin and Strauss),^9^ was not used as the aim was to identify emerging themes.

AL and JS manually coded the data independently in NVivo 14 (Lumivero; Denver, Colorado USA) using an open coding technique.^10^ AL and JS then compared codes. JS was able to provide professional insight into some of the participants’ responses for clarity whilst the fact that AL was not a pharmacist reduced the risk of bias. Through discussion, a single set of codes was derived and then condensed into a smaller number of categories (sub-themes) and finally into key themes.^10,11^ EM and KW provided feedback on the allocation of codes to data, and also on the coding tree, with revisions made through team discussions.

## Observations

**Figure 1.**
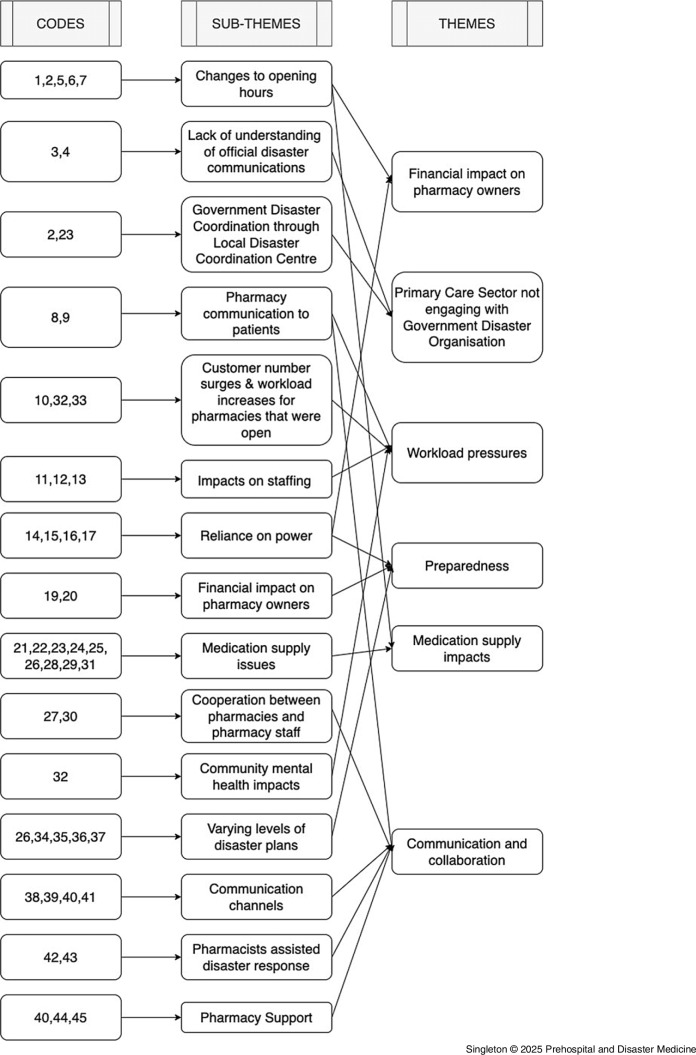
Coding Tree for the Thematic Analysis of Interview Data.

The thematic analysis yielded six themes – “financial impact on pharmacy owners,” “engagement with Local Disaster Coordination Center (LDCC) important,” “workload pressures,” “preparedness,” “medication supply impacts,” and “communication and collaboration” (Figure 1). Themes and their representative quotations are presented in Table 1 (codes and their representative quotations are presented in Supplementary Material).

**Table 1.** Themes and Representative Quotations

Themes	Representative Quotations
**Financial Impact on Pharmacy Owners**	“…the other thing we really learned was you’re at the mercy of your landlord. The owner of the shopping center and their insurance company were unwilling to pay for a generator so when we did open and Woolworths opened, they wouldn’t even pay to have a generator to put lights at the entrances of the shopping center or anything or security. So, we had to provide our own security because obviously automatic doors and everything like that are gone and we have to provide our own security, our own lights, our own fans. They tried to say it wasn’t safe to open but, in the end, when it got down to it, there weren’t that many pharmacies open so we just opened. But they couldn’t physically stop us from opening.” - [N212] “Several pharmacies closed as precautionary measures. They weren’t at risk but there’s a misperception about what the disaster management messages are. And when it goes out to please stay off the roads unless you’re essential services, a lot of the pharmacies shut. A lot of the GP practices shut…… they’re essential services.” - [C232] “On a couple of days, we’d close earlier than we would have usually - at least two hours early.” [L271] - “…there was a lot of disruption early because all of the pharmacies shut when they didn’t really have to.” - [C232] “The pharmacy itself wasn’t completely flooded. There was no access to the pharmacy. All the surrounding roads were closed off.” - [N229] “…we didn’t have power for five days so we ran off a generator. We didn’t have fridge stock, anything like that. We’re in a shopping center but the shopping center’s owners wouldn’t pay for a generator. We had to buy a generator … to have enough power to dispense. But we couldn’t power a fridge or lights or anything like that.” - [N212] “We ended up losing some vaccines that couldn’t go anywhere basically. So, it was a choice of what we wanted to keep because, in the end, everyone around town started losing power and not everyone had the generator.” - [N2212] “It was just the joys of learning to work with only one dispense computer, one printer, and one label printer when you’ve got no internet or lights or fans or anything. So yeah, it was just a learning curve…. It would have been, I don’t know, between thirty and forty degrees every time we were working in there. So yeah, it wasn’t ideal.” - [N212] “…how confident are you this person is going to come back? And to be frank, a lot of them didn’t bring the script back. Often the patients didn’t have money. You do it as an owing and in that say, hey, when things settle down come back to us. And they didn’t. But that’s okay. So, the pharmacy is wearing that cost.” - [C232] “They weren’t all reconciled. We don’t have a lot outstanding - half a dozen patients possibly. We also found it very difficult that, after it had all settled down and we were able to identify doctors’ surgeries, and we tried to contact the doctors’ surgeries for some of them. Surgeries were not helpful at all. They wouldn’t even provide us with patient phone numbers so we could chase these people up for them to get the opportunity to go to the doctor to try and get prescriptions. They wouldn’t provide us with patient information. They said it was a confidentiality issue.” - [Y52] “We also supplied them with hand sanitizer, bandages, basic first aid, stuff like that. Hydralyte, things like that to them to provide to volunteers and people that were coming through seeking assistance.” [Y52]
**Engagement with LDCC Important**	“…we were keeping it open based on advice from the Local Disaster Coordination Center. But it was in a risky area. So, we – but in consultation with the emergency Management, we were trying to keep it open as long as possible, but we were forced to shut that. So, it closed late on Saturday.” - [C232] “There’s a misperception about what the disaster management messages are. And when it goes out, that please stay off the roads unless you’re essential services, a lot of the pharmacies shut. A lot of the GP practices shut.” - [C232] “…there’s usually emergency services roadblocks, and then we would discuss with the emergency services, well, we are in communications with a patient down in this area. What would you like us to do? Nine times out of ten, the emergency services say, give us the medication and we’ll take it to them. They flew them out. Army helicopters or a QAS helicopter. They don’t like boating across flood waters. It’s all helicopter.” - [C232] “To deliver Webster-packs, because a whole heap of our customers where we were, they’re in low lying parts that we deliver to – couldn’t drive to. So, the SES ended up delivering Webster-packs by boat to some people.” - [N212]
**Workload Pressures**	“…we took on all the extra patients from our Aboriginal and Islander health service pharmacy, and the [XX] pharmacy.” - [C232] “There was an influx of people. We were crazy. Crazy busy for that period of time. We had our script – script numbers for instance would have doubled on those days, and we were running on very limited staff.” - [L271] “…we got traffic from all over because we were getting people who had been displaced and were staying in evacuation centers coming in as well.” - [Y52] “The people who were there managed, but for instance, script wait times might have been half an hour. Half an hour upwards in periods. The public were fairly forgiving during that time. A lot of them had driven all over town try and find a pharmacy that was open, but it wasn’t ideal.” - [L271] “Maybe a week after the floods though, we did have an increase. We actually took on a number of customers from another pharmacy that unfortunately flooded, and we managed their patients for a number of months while they were getting re-fitted.” - [A25] “The [XX] pharmacy has quite a significant packing role for Queensland Health. It packs for three aged care facilities, Townsville Corrections, and Palm Island. So, we had to keep those services operating from [YY] site… went from packing by robot to packing by hand for 1,500 packs a week.” - [C232]
**Preparedness**	“…when you’re in a shopping center that’s about four feet above where any water would usually get to, you don’t have flood insurance.” - [N212] “XX pharmacy is a large supplier for opioid replacement therapy … We didn’t know it at the time, but we had closed it in consultation with the Local Disaster Coordination Center and in consultation with Townsville Hospital. We said, do you want us to evacuate the medicine? At the time they said no, because none of us realized, I think, the significance. But … less than 24 hours later, the hospital realized how many of our patients they were now required to service, and they asked us to go back, and we were able to recover the medicine required and … and recommence providing that service from [XX] site.” - [C232] “No, we don’t have a disaster plan.” - [L271] “Not really. The plan was that if it ever happened, because we’ve had cyclones and everything up here before, we just as soon as we know we’re going to lose power, we get all the fridge stock basically. But that’s about it. There’s no actual plan. Because we didn’t have our own generator here, we basically had to run a giant extension cord out to power everything. It was kind of play it by ear and just see what happens.” - [N212] “…overall had a very clear North Queensland cyclone focus. So, what was very new for us was a flood event. It had a few key features that are different because of the longevity of the event. Our experience with cyclones is there’s a lot of planning and consideration leading up to the event. You get a fairly good warning period. A much longer warning period for a cyclone than what you get for a flood…… The response phase in a cyclone is six to twelve hours. Our response phase in a flood was thirteen days. So, it was very different. A whole lot of different planning considerations.” - [C232]
**Medication Supply Impacts**	“The supply of medication to evacuation centers became quite significant as well. It was new and disruptive…people turn up to evacuation centers that don’t have their scripts. They don’t know what their medications are.” - [C232] “Our delivery service was impeded. So, we couldn’t do all our normal deliveries.” - [Y52] “A couple of them were true emergency supplies, but then no one had records for that patient. We were relying on the patient – the patient had brought in a box, or a box that was damaged by water, that kind of thing. Or a script that had basically – the ink of the script had been washed away.” - [N212] “I don’t think the three-day emergency supply rule is efficient in these types of events. I think we were fortunate that for most people, three days saw them through the worst of it so they could access their usual pharmacy by that point. But there were cases where pharmacies were closed for months because they were drastically affected by the floods…. those patients …then had to go and find sometimes a different doctor and then relocate to another pharmacy. That’s probably a situation where they could require a… disaster emergency supply where they maybe get a larger supply allowed to them.” - [L271] “API wouldn’t deliver. Sigma wouldn’t deliver most of the time and Symbion …it ended up that we would just message the warehouse manager what we needed and he would bring it to us if he could.” - [N212] “We found we got short of stock. We just had to prioritize really. We ran short of a lot of stuff. We keep a lot of stock anyway, and then the other problem was that all the other pharmacies started sending people to us because we had a generator and we were still open. So, we started going through lines that we’d normally only do one a month to suddenly doing three or four. We could get it through Symbion and have it the next day, but there would have been people that went without stuff because we had to try and find it somewhere.”- [N212]
**Communication and Collaboration**	“Our center management stayed in good contact with everyone, because we’re in a shopping center. To make sure that everyone was aware of what was going on… So, we were kept in really good touch with everything going on.” - [A25] “Once we had left the pharmacy, we couldn’t get updates from anyone as to whether our store had flooded or not. We knew there was no power. We knew there were no phone lines … it all lost power… the only way we found out that the store hadn’t gone under water was because the owner actually walked in through about two feet of water to get to the store and see.” - [N212] “We just kept checking the Bureau of Meteorology. We didn’t receive any calls from the Department of Health, emergency services, or the disaster management team.” - [L271] “…the owner here, she’s on a number of committees and she’s on the Federal MP’s health team, … getting information from a lot of the disaster briefings, which they were then able to relay very quickly back to the staff group, through our Facebook staff closed group. We’d get alerts and updates through that.” - [Y52] “We did comment or post on their (LDCC) Facebook page, and also on a couple of local radio Facebook pages - that seemed to get word out that we were one of very few pharmacies actually open.” - [L271] “…because we’re a relatively small community (pharmacists), we have a Facebook group chat. There’s someone from every pharmacy in it… pharmacists were just messaging on there saying is anyone able to work this shift at this center….” - [N212]

### Financial Impacts

Understandably, the floods caused financial impacts for pharmacy owners of affected pharmacies that were forced to close. Some closures were precautionary measures which may not have been necessary. One pharmacy’s landlord did not provide support to help the pharmacy to remain open. For those pharmacies that were open, some patients were unable to pay, and many patients who were supplied medication without a prescription never returned to the pharmacy with the prescription. This meant the pharmacies could not claim reimbursement and were impacted financially.

### Engagement with LDCC Important

Those participants who reported their pharmacy owners engaged with, and took advice from, the LDCC appeared to be better informed of flood developments.

### Workload Pressures

Participants reported increased workload pressures from supplying medications to people whose regular pharmacy was closed, reduced staff numbers, and austere working conditions.

### Preparedness

Pharmacies (and the community) were under-prepared for this flooding event. One pharmacy, which was able to stay open, had no power and had to obtain a generator at a time when many other residents and business owners were also trying to source a generator. Ensuing issues with continuity of supply for Opioid Replacement Therapy (ORT) clients also highlighted a potentially overlooked patient group in disaster events. With regards to pharmacies having a documented disaster plan, responses varied from having no plan to a plan that was developed with a focus on cyclones rather than a flooding event.

### Medication Supply Impacts

With pharmacies having to reduce hours or close completely, medication supply to patients was impacted, particularly for the large number of patients receiving Dose Administration Aids (DAA) and for patients whose pharmacies retained their prescriptions. Sometimes, medication supply was impeded because the flooded river prevented people accessing a pharmacy. People whose homes were flooded also had medication supply issues. If they were unable to access their regular pharmacy, then obtaining their medications became a challenge, particularly if they didn’t know the names of the drugs they were taking. Participants reported that medication supplies in these circumstances were through Australia’s three-day emergency supply legislation. Some medication were supplied as “owings” where the pharmacy lends the patient a Pharmaceutical Benefits Scheme (PBS) quantity of the medication (usually one month’s supply) with the proviso that the patient returns to the pharmacy with the prescription as soon as they can. Many of these patients presented to evacuation centers with no medications, no prescriptions, and no idea of what they were taking.

The loss of power also impacted medication continuity. Patients lost their refrigerated medications and pharmacies that lost power had to store medications requiring refrigeration off-site. For those pharmacies who still had power or had a generator, My Health Record (Australia’s national digital health record platform) helped pharmacists obtain medication histories for those patients who presented to the pharmacy without prescriptions. Deliveries of medications from the three pharmacy wholesalers were also interrupted, and one of the pharmacies that remained open reported stock shortages. The defense services and emergency services (coordinated through the LDCC) assisted pharmacies in sending medications to locations outside of Townsville. Importantly, pharmacy staff worked hard with stakeholders to ensure there was not a complete breakdown (albeit some delays) in critical medication supplies.

### Communication and Collaboration

Facebook (Meta; Menlo Park, California USA) groups were the main communication platform. Some participants spoke of collaborating with other pharmacies to consolidate resources at sites that were open, and some pharmacists whose regular place of employment had closed helped in pharmacies that remained open. Pharmacists also helped at the emergency evacuation centers and provided support to the LDCC.

## Analysis

Whilst it was apparent that the Townsville community was unprepared for this flood event, this study highlighted that pharmacies communicated and collaborated with each other to ensure the community was able to access medications and other health needs in a timely fashion. Despite the increased work pressures and the austere working conditions (no lighting or air conditioning in pharmacies running on generators), pharmacy staff were able to avoid a complete breakdown of medication supply.

The key themes that emerged from the interview data (Figure 1) have also emerged from research on the impacts of other disaster events in Australia on community pharmacy operations.^7,8,12,13^ During this flooding event, information gathering appeared to be passive rather than proactive; in many cases, participants appeared to be waiting for “someone in authority” to pass on information about the event. This corroborates the findings of Safnuk, et al who theorized that pharmacists revert to a “novice” level of understanding in disaster situations and require explicit instructions.^14^ Only two participants appeared to understand local disaster management processes and were able to discuss the role of the LDCC and Primary Health Network (PHN). This lack of engagement may have resulted in some pharmacies and General Practitioner clinics closing prematurely due to the owners not understanding what constitutes an “essential service” in disaster messages. These closures have financial impacts for owners. Other financial losses, such as through medication supplies to patients who never returned with a prescription or with money, is an on-going issue for pharmacies during disaster events and has been reported in other disaster research studies.^7,8^

Preparedness also has financial implications. Power outages are highly probable in any disaster event, and purchasing a generator outside a disaster event when demand is much lower could save money, potentially reduce the risk of losing medications which must be stored in a refrigerator, and allow the pharmacy to keep operating. Preparedness and preparedness behaviors in Australian pharmacists has been found previously to be low-to-moderate and low, respectively.^15^ This is an important point; for community pharmacies to be operational during a disaster, they must be staffed by prepared pharmacists.^15-18^ An individual’s preparedness is influenced by numerous variables. These include age (older participants report higher levels of preparedness),^19^ self-efficacy for preparedness,^19-22^ perceived severity of the disaster (the greater the perception of the severity of the disaster, the lower the preparedness),^15,19^ and knowledge and skills.^15^ Context is also important. To have prepared community pharmacies, there needs to be prepared pharmacists, and an individual pharmacist’s preparedness is linked to the preparedness of others in their workplace.^15^

One pharmacist commented that patients with chronic conditions failed to stock up on on-going medications ahead of the flood event. This comment highlights the need for research exploring the concerns of people living with chronic conditions when they receive a disaster event warning. These patients may have reduced physical capacity to organize extra supplies and prescriptions and may have to rely on others. Another patient group affected by disaster events is the ORT program clients because both prescribers and clients can be hard to contact by pharmacists. Staged supply arrangements (where the client comes into the pharmacy daily to collect their opioid dose) may need to be changed so the client receives a number of take-away doses for the duration of the disaster event. However, this and another study found that prescribing clinics often close without contacting pharmacies or clients to make alternative supply arrangements for patients.^23^

There were several limitations to this study. Firstly, the small number of participants risked the data potentially not reflecting a diversity of opinions, making it difficult to determine if data saturation was reached. Secondly, the data were collected in 2019 with the analysis and write up delayed by the COVID-19 pandemic. Lastly, financial data pertaining to the financial loss to pharmacies and hard data on patient days without medication are not collated currently and/or publicly available.

This research highlights a critical need for both improved communication at the public-private interface and greater disaster preparedness among pharmacists to improve the primary care sector’s response. This need could be addressed by including disaster preparedness in pharmacy Continuing Professional Development (CPD) programs. Pharmacy CPD educators could use the International Pharmaceutical Federation (FIP; The Hague, The Netherlands)’s “Global Humanitarian Competency Framework”^24^ as a guide. This framework has four domains, two of which align with this study’s findings. The “Pharmaceutical Public Health” domain has a population focus with two competencies – “health assessment” and “health, medications information, and advice.” The “Organization and Management” domain comprises “budget,” “human resources management,” “improvement of service,” “procurement,” “supply chain management,” and “workplace management” competencies. These competencies are also applicable to pharmacist preparedness in other natural weather events nationally and globally.

## Supporting information

Singleton et al. supplementary material 1 Singleton et al. supplementary material

Singleton et al. supplementary material 2 Singleton et al. supplementary material

Singleton et al. supplementary material 3 Singleton et al. supplementary material

## Supplementary Materials

To view supplementary material for this article, please visit https://doi.org/10.1017/S1049023X25101301
