# Supplementary material for: An Exploration of the Impacts of the 2019 Floods in Townsville, Australia on Community Pharmacy Operations
Source: Prehosp Disaster Med. 2025 Aug 27;40(4):232–8. doi: 10.1017/S1049023X25101301 (PMC12809201; doi:10.1017/S1049023X25101301)
Supplement: Singleton et al. supplementary material 1 — Singleton et al. supplementary material [file S1049023X25101301sup001.pdf]

Unique Identifier: ________________

Project: Impact of Recent Disasters on Community Pharmacy Services in Australia

Semi-structured Interview Questions

These interviews are semi-structured and depending on the responses of the participant the interviewer may choose to pursue further a particular line of discussion. The questions below are simply a guide. The interview is expected to take approximately 20 minutes.

1. During the period of the disaster [insert description] can you recall if the day-to-day operations of the pharmacy were in any way different to normal? (e.g. was the pharmacy under threat, did it experience power blackouts, or have to close?)

If 'no' jump to Q4

If 'yes' to Q1:

1a) Did the pharmacy have a business disaster plan or procedures in place (e.g. generator, offsite data backup) prior to the disaster?

2. During the disaster [insert description] was pharmacy staffing an issue? Were staff able to get to and from work, or did issues arise as people dealt with risk to their own personal property and ensuring family members were safe?

3. During the period of the disaster [insert description], were the provision of regular pharmacy services impeded?

    (How were the pharmacy services impeded?)

    (Were there logistical or operational issues? e.g. power outages, unable to supply medications because suppliers couldn't deliver stock)

If 'yes' to Q3:

3a) Can you recall the length of delays patients experienced in receiving appropriate care as a result of reduced pharmacy services?

4. During the period of the disaster [insert description], were you asked to supply medications to any patients who were unable to present scripts to you? (e.g. because they had lost all their possessions, did not take scripts with them when they were evacuated, or kept their scripts at another pharmacy that was closed).

(Were there any regulatory or ethical issues that arose as a result of this request?)
(Were they able to be resolved satisfactorily?)

5. During the period of the disaster [insert description], were you kept up-to-date with information on the status of the disaster [insert description]?

6. Did you provide information to emergency services / command centre of the pharmacy's opening hours / stock available / pharmacy services still operating?

If 'no' to Q6:
6a) Would you know who to contact to provide this information in another event?

7. During the period of the disaster [insert description], were you provided with any support from the Department of Health or professional pharmacy bodies to assist you in running your pharmacy and helping the community with medications supply?

8. As we face the prospect of an increase in the frequency and severity of extreme weather events, do you have any suggestions about how the pharmacy profession could prepare to ensure continuity of medication supply to patients?
