# Supplementary material for: An Exploration of the Impacts of the 2019 Floods in Townsville, Australia on Community Pharmacy Operations
Source: Prehosp Disaster Med. 2025 Aug 27;40(4):232–8. doi: 10.1017/S1049023X25101301 (PMC12809201; doi:10.1017/S1049023X25101301)
Supplement: Singleton et al. supplementary material 2 — Singleton et al. supplementary material [file S1049023X25101301sup002.pdf]

Code Number	Code	Representative Quotations
1	Landlord Control	*"…the other thing we really learned was you're at the mercy of your landlord. The owner of the shopping centre and their insurance company were unwilling to pay for a generator so when we did open and Woolworths opened they wouldn't even pay to have a generator to put lights at the entrances of the shopping centre or anything or security. So we had to provide our own security because obviously automatic doors and everything like that are gone and we have to provide our own security, our own lights, our own fans. They tried to say it wasn't safe to open but in the end when it got down to it there weren't that many pharmacies open so we just opened. But they couldn't physically stop us from opening." [N212]*
2	Pharmacy opening hours based on advice from Local Disaster Coordination Centre	*"…we were keeping it open based on advice from the Local Disaster Co-ordination Centre. But it was in a risky area. So we – but in consultation with the emergency management we were trying to keep it open as long as possible, but we were forced to shut that. So it closed late on Saturday." [C232]*
3	Some pharmacies and GP practices closed prematurely	*"Several pharmacies closed as precautionary measures. They weren't at risk but there's a - we're doing a lot of promotion in Townsville now. There's a misperception about what the disaster management messages are.And when it goes out, that please stay off the roads unless you're essential services, a lot of the pharmacies shut. A lot of the GP practices shut." [C232]; "One shut very early as a precautionary measure. It was co-located with a medical centre that shut." [C232] ;*<br>*"On a couple of days we'd closed earlier than we would have usually - at least two hours early." [L271] ; "…there was a lot of disruption early because all of the pharmacies shut when they didn't really have to." [C232]*
4	Lack of understanding of what constitutes an essential service	*"A GP practice in a disaster situation is an essential service. A pharmacy is an essential service. A lot of pharmacies even here in Townsville didn't know that." [C232]*
5	Pharmacy closed because flood waters cut off access	*"The pharmacy itself wasn't completely flooded. There was no access to the pharmacy. All the surrounding roads were closed off." [N229]*
6	Pharmacy remained open	*"The pharmacy itself wasn't completely flooded. There was no access to the pharmacy. All the surrounding roads were closed off." [N229]*
7	Pharmacy opened additional hours	*"Actually they did open up on a Sunday for a period of time, which we don't usually trade on to help one of those disaster relief places bring busloads of people here." [Y52]*
8	Notified customers of services' disruption	*"We have a script management system where we can send out a bulk text message to our customers to let them know that we would be unable to operate, and if they needed things, where to go." [A25]*
9	Provided information on where to access alternative pharmacy options	*"…if they needed things, where to go. We sent them through a list of Allied Health and open pharmacies as well." [A25]*
10	Pharmacies that were open experienced patient surge	*"…we took on all the extra patients from our Aboriginal and Islander health service pharmacy, and the [XX] pharmacy" [C232] ; "*<br>*There was an influx of people. We were crazy. Crazy busy for that period of time. We had our script – script numbers for instance would have doubled on those days, and we were running on very limited staff." [L271] ; "…*<br>*we got traffic from all over because we were getting people who had been displaced and were staying in evacuation centres coming in as well." [Y52] ; "*<br>*The people who were there managed, but for instance script wait times might have been half an hour. Half an hour upwards in periods. The public were fairly forgiving during that time. A lot of them had driven all over town try and find a pharmacy that was open but It wasn't ideal." [L271] ; "*<br>*Maybe a week after the floods though we did have an increase. We actually took on a number of customers from another pharmacy that unfortunately flooded,*

		and we managed their patients for a number of months while they were getting re-fitted.” [A25] ; “… two pharmacies were completely destroyed and another two got flooded. So their patients, even when the pharmacies started opening again, their patients had to go somewhere.” [C232]
11	Owner & managing Px absent at time of flooding	*“Our owner and managing pharmacist were away.” [A25]*
12	Impacts on staffing	*So, there was a period of time where we did not have access to the pharmacy. We couldn’t get in – physically into the centre.” [A25] ; “I had a number of staff who couldn’t make it to work due to roads closing.” [L271] ; “Yeah a massive one. From the Thursday when it had already been a couple of days of heavy rain, this was before the massive flooding when they opened the dam gates , the two days prior to that it was just me [de-identified] another pharmacist, and the dispense tech. We were the only ones willing to go there. Other people were either flooded in or weren’t willing to drive. So rather than running with – we usually run with about eight staff a day, depending on what we’re doing, it was one or two of us.” [N212] ; “So one of my pharmacists actually lost her home to the floods and wasn’t able to come in . And obviously without a pharmacist I can’t open. So that did impact it . I was lucky enough to be able to get a locum for a couple of days. But we did have to close for several days post flood – post and during flood.” [A25] ; “So we had no shortage of people trying to make their way to work. We had people turning up, their houses had been flooded. They’re going, well my house is destroyed, I may as well come to work. We got – they come to work in board shorts, thongs, and a singlet, but sadly because it’s all they’ve got. Then you’re going, man I can’t have you coming to work in thongs. Wading through a foot worth of water in thongs. That’s an occupational health and safety risk, and we need to be conscious of that. I really appreciate that you’re volunteering, but we need to volunteer in a rational and safe way.” [C232] ; “Staff weren’t able to get to and from work. Probably half of the staff . And schools and day cares asked their parents to come and collect their children so staff had to leave to collect them.” [N229] ; “We were able to stay adequately staffed. So I think there’s seven pharmacists across the three stores. At least two were displaced . Other than that we had enough staff to keep running the store and front shop as well. We did have the [XX] pharmacy staff to help, and it was generally the [YY] staff running walk-ins and dispensing all the scripts and the [XX] staff basically set up a temporary Webster-packing station in the back of the store and they made sure their Webster-packing component of things was running over because they have a very large Webster-packing clientele.” [Y52]*
13	Staff from unaffected pharmacies assisted in operations	*“We had no shortage of volunteers. Employees volunteering to come and help because the whole community was banding together.” [C232]*
14	Loss of power affected operations	*“…we didn’t have power for five days so we ran off a generator. We didn’t have fridge stock, anything like that. We’re in a shopping centre but the shopping centre’s owners wouldn’t pay for a generator. We had to buy a generator and use our own generator to have enough power to dispense. But we couldn’t power a fridge or lights or anything like that.” [N212] ; “We ended up losing some vaccines that couldn’t go anywhere basically. So it was a choice of what we wanted to keep because in the end everyone around town started losing power and not everyone had the generator.” [N2212] ; “It was just the joys of learning to work with only one dispense computer, one printer and one label printer when you’ve got no internet or lights or fans or anything. So yeah, it was just a learning curve.” [N212] ; “ if you’re talking about ideal storage of like, non-fridge lines in the shopping centre that we were working within we’re not near the entrance. It would have been, I don’t know, between thirty and forty degrees every time we were working in there. So yeah, it wasn’t ideal.” [N212]*
15	Fridge stock taken pre-emptively off-site	*“When we knew we were going to lose power all our 'frig lines got taken off site…. It was basically a day delay. [If customers] brought in a script for fridge stock we’d have it to them – if they were desperate we’d get it to them that night. If not, they couldn’t get it until the next day basically.” [N212]*

16	Off-site pharmacy fridge stock storage caused patient delays	*"It was basically a day delay. They brought in a script for fridge stock. We'd have it to them – if they were desperate we'd get it to them that night. If not, they couldn't get it until the next day basically." [N212]*
17	Patients lost refrigerated medications	*"Everyone around us had no power for some people up to two or three weeks. Every patient lost their fridge stock as well." [N212]*
18	Pharmacy resources consolidated into a single site	*..all of the patient files and packs from the other two pharmacies were evacuated to the third pharmacy so we could provide continuative care....we were able to keep packing [webster packs] from the third site. [C232] ; "…we just pooled all our resources down here . And the [XX] store was closed I think probably about four – minimum of four, maybe five days I think." [Y52]*
19	No flood insurance	*"…when you're in a shopping centre that's about four feet above where any water would usually get to, you don't have flood insurance." [N212]*
20	Financial impacts on Pharmacies	*"…how confident are you this person is going to come back? And to be frank, a lot of them didn't bring the script back. Often the patients didn't have money. You do it as an owing and in that say, hey when things settle down come back to us. And they didn't. But that's okay. You just have to make sure people are safe. So the pharmacy is wearing that cost." [C232] ; "They weren't all reconciled. We don't have a lot outstanding - half a dozen patients possibly. We also found it very difficult that, after it had all settled down and we were able to identify doctors' surgeries and we tried to contact the doctors' surgeries for some of them. Surgeries were not helpful at all. They wouldn't even provide us with patient phone numbers so we could chase these people up for them to get the opportunity to go to the doctor to try and get prescriptions. They wouldn't provide us with patient information. They said it was a confidentiality issue." [Y52] ; "We also supplied them with hand sanitiser, bandages, basic first aid stuff like that. Hydralyte, things like that to them to provide to volunteers and people that were coming through seeking assistance. I believe our community care account funded it but I'm not 100% on that." [Y52]*
21	Patients arrived at Evacuation Centres without Rx or meds	*"The supply of medication to evacuation centres became quite significant as well. It was new and disruptive...people turn up to evacuation centres that don't have their scripts. They don't know what their medications are." [C232]*
22	Patient RX issues with pharmacy closures	*"So there were a few of our DAA customers and script on file customers that did need to go back to their doctors, or back to a doctor and get their medications that way. If they'd run out while we were closed." [A25] ; "In rare cases where, for instance, there was a pharmacy that actually flooded and patients had scripts at that pharmacy that were left there but they couldn't access. So we would phone the pharmacists that were there that gave us mobile numbers and said if they knew the patients or could access their dispensing software remotely to get records. In worst case scenarios, there was a couple cases where patients that had brought in boxes of medication where they didn't have any more. They didn't have scripts. I'd give them an emergency supply." [C232] ; "<br>It was a mixture of three day emergency supplies and owings. It was a combination I suppose of it. The pharmacists definitely made an assessment based on, was the patient known to us at that pharmacy, then we're more likely to do it as an owing. Or if the patient was completely unknown to us then you'd probably do it as an emergency dose. So I think that's the factor about, how confident are you this person is going to come back? And to be frank, a lot of them didn't bring the script back." [C232] ; "<br>We managed to get them the scripts back. Doctors came through for us and we got a whole heap of scripts dropped off. There were a couple of other ones that we had to follow up. So the doctors that worked in the disaster centres.. just said after it follow me up and tell me what you need. It took a couple of weeks, ..Within a month everything was kind of fixed." [N212]*
23	Medicines delivered by alternative means – defence forces, SES, ferries	*"…we had to get the packs to locations like Charters Towers which was cut off by the Burdekin River, and also out to Palm Island, and moving around the area locally. So we had deliveries conducted by our in-house delivery drivers in our own delivery van, by defence helicopter out to Charters Towers, by ambulance helicopter, and by ferry crews. The airport – normally we use air freight to go to Palm Island but the sheer volume of rain was periodically shutting the airport. So the ferries were still operating, and we got the medicine out there by ferry."*

		[C232] ; "…there's usually emergency services roadblocks, and then we would discuss with the emergency services, well, we are in communications with a patient down in this area. What would you like us to do? Nine times out of ten the emergency services say, give us the medication and we'll take it to them." [C232] ; "They flew them out. Army helicopters or a QAS helicopter. They don't like boating across flood waters. It's all helicopter." [C232] ; "To deliver Webster-paks, because a whole heap of our customers where we were they're in low lying parts that we deliver to – couldn't drive to. So the SES ended up delivering Webster-paks by boat to some people." [N212]
24	Flood directly impacted patients' medication access	*"…the actual physical river isolated people from alternative pharmacies. The two pharmacies that got destroyed for example were on the south-eastern side of the river, and there were no other pharmacies on the south-eastern side of the river around those suburbs. So actually getting them medication became a challenge." [C232] ; "Our delivery service was impeded. So we couldn't do all our normal deliveries." [Y52] ; "A couple of them were true emergency supplies but then no one had records for that patient. We were relying on the patient – the patient had brought in a box, or a box that was damaged by water, that kind of thing. Or a script that had basically – the ink of the script had been washed away." [N212]*
25	Limitations with 3-Day Emergency Supply Rule	*"I don't think the three-day emergency supply rule is efficient in these types of events. I think we were fortunate that for most people three days saw them through the worst of it so they could access their usual pharmacy by that point. But there was cases like I mentioned where pharmacies were closed for months because they were drastically affected by the floods. So for those patients they then had to go and find sometimes a different doctor and then relocate to another pharmacy. That's probably a situation where they could require a, I don't know, a disaster emergency supply where they maybe get a larger supply allowed to them." [L271]*
26	Failure to estimate extent of medication requirements of ORT patients	*"XX pharmacy is the largest supplier for opioid replacement therapy outside of Townsville Hospital. We didn't know it at the time, but we had closed it in consultation with the Local Disaster Co-ordination Centre and in consultation with Townsville Hospital. We said, do you want us to evacuate the medicine? At the time they said no, because none of us realised, I think, the significance. But on Sunday, so less than 24 hours later, the hospital realised how many of our patients they were now required to service, and they asked us to go back, and we were able to recover the medicine required and secure it at the [Garbutt] site and recommence providing that service from [XX] site." [C232]*
27	ORT patient supplies and RX transferred to another pharmacy in pharmacy group	*"…we also actually dosed some other methadone patients from other pharmacies that were closed." [Y52]*
28	Wholesaler deliveries interrupted	*"API wouldn't deliver. Sigma wouldn't deliver most of the time and Symbion who I guess is considered our main wholesaler, it ended up that we would just message the warehouse manager what we needed and he would bring it to us if he could." [N212]*
29	Stock shortages	*"We found we got short of stock. We just had to prioritise really. We ran short of a lot of stuff. We keep a lot of stock anyway, and then the other problem was that all the other pharmacies started sending people to us because we had a generator and we were still open. So we started going through lines that we'd normally only do one a month to suddenly doing three or four . If we had time we could get it through Symbion and have it the next day, but there would have been people that went without stuff because we had to try and find it somewhere." [N212]*
30	When pharmacies with DAA tenders to facilities are forced to close, workload shifts to other pharmacies	*"The [XX] pharmacy has quite a significant packing role for Queensland Health. It packs for three aged care facilities, Townsville Corrections, and Palm Island. So we had to keep those services operating from [YY] site. So we moved that responsibility to [Garbutt]. Shifted from packing by robot to packing by hand for 1,500 packs a week." [C232]*
31	My Health Record assisted medication reconciliation	*"…people turn up to evacuation centres that don't have their scripts. They don't know what their medications are and fortunately with tools like My Health Record it doesn't matter anymore. We were able to successfully and safely quite*

		accurately determine their medication profiles. My Health Record definitely resolved and smoothed out a lot of issues. Especially for those displaced people." [C232] ; "It was more owings. I think we might have done if it was like a medication or potential abuse or something we weren't sure on and we would – it was more like a goodwill and someone said I don't have my anti-depressant kind of thing. We had nothing. Depending on what it was, it was those cases tended to be an emergency supply. But if we could get onto the My Health Record and see that they were a regular medication and that sort of thing, they tended to be an owing." [Y52]
32	Increase in mental health medication supplies	*"Potentially probably more stress related dispensings – diazepams and the anxiety sort of stuff. So increases in those sorts of dispensings for sure." [A25]*
33	Increased First Aid Requests	*"There was definitely an increase in the number of people that came in that were requiring First Aid help." [L271]*
34	No Disaster Plan	*"No, we don't have a plan." [L271] ; "Not really. The plan was that if it ever happened, because we've had cyclones and everything up here before, we just as soon as we know we're going to lose power we get all the fridge stock basically. But that's about it. There's no actual plan. Because we didn't have our own generator here we basically had to run a giant extension cord out to power everything. It was kind of play it by ear and just see what happens." [N212]*
35	Disaster plans had cyclone focus	*"…overall had a very clear North Queensland cyclone focus. So what was very new for us was a flood event. It had a few key features that are different because of the longevity of the event. Our experience with cyclones are there's a lot of planning and consideration leading up to the event. You get a fairly good warning period. A much longer warning period for a cyclone than what you get for a flood." [C232] ; "With a flood, it just goes on, and on, and on. The response phase in a cyclone is six to twelve hours. Our response phase in a flood was thirteen days. So it was very different. A whole lot of different planning considerations." [C232] ;*
36	Pharmacy had disaster plan	*"Yes they did." [N229] ; "Yes they did." [Y52] ; "The plan was – safety to us as individuals was priority, so that if we didn't feel safe coming out to work or being on the road for whatever reason then that took priority. We could stay home if we didn't want to come to work . Then it was – we were getting regular updates through our pharmacy's social media page which staff access. That was, like, the state of things in Townsville. What was open, who was trading. That sort of thing. Our bosses worked very closely with the Townsville disaster committee sort of thing so they were getting up to date information by that and transferring it onto us." [Y52] ;*<br>*"Yes. – and it's been tested – it's been designed in consultation with our Local Disaster Manager who runs the Local Disaster Co-ordination Centre and also Emergency Management Queensland." [C232]*
37	Staff unsure if pharmacy had a Disaster Plan	*"…me and [Luke], the pharmacist, were the only regular staff members here. So it was – we really didn't get to, I guess, go through it before everything got crazy . And then he was away so it was kind of just playing everything by ear." [A25]*
38	Communication on Disaster Status	*"Our centre management stayed in good contact with everyone, because we're in a shopping centre. To make sure that everyone was aware of what was going on, what timelines were, what things were operating, what wasn't. So we were kept in really good touch with everything going on." [A25] ; "With regards to the flood waters, I think it was just the Bureau of Meteorology." [N229] ; "In the Local Disaster Co-ordination Centres they've got operations cells, - they've got health cells. And that's where we tap into – also your Primary Health Networks. Our PHN (Northern Queensland PHN) is tapped into that health cell of the Local Disaster Co-ordination Centre as well. So then that health cell has got the PHN and HHS all integrated." [C232] ; "Emergency Services Facebook page plus the radio." [C232] ; "Once we had left the pharmacy we couldn't get updates form anyone as to whether our store had flooded or not. We knew there was no power. We knew there were no phone lines or anything because it's all NBN stuff and it all lost power . But the only way we found out that the store hadn't gone under water was because the owner actually walked in through about two feet of water to get to the store and see." [N212] ; "We just kept checking the Bureau of Meteorology. We didn't receive any calls from the Department of Health, emergency services or the disaster management team. Not to my*

		knowledge." [L271] ; "…the owner here, she's on a number of committees and she's on the Federal MP's health team, and they were getting information from a lot of the disaster briefings which they were then able to relay very quickly back to the staff group, which would generally be through our Facebook staff closed group . We'd get alerts and updates through that." [Y52] ; *"Sometimes, when it was just a co-ordination measure, like about our particular job, we just used email and SMS and phone call for clarity.... We tried to use phone ...to clarify really confusing or new things. With phone call as a last resort because the phone lines were just so busy . Often you just pose the question by text and wait." [C232] ; "We didn't communicate our status to emergency services directly, but only just to the pharmacy's Facebook page. We could tell people were accessing it." [N229]*
39	Communication through Facebook	*"…getting information from a lot of the disaster briefings which they were then able to relay very quickly back to the staff group, which would generally be through our Facebook staff closed group." [Y52] ; "I managed that through our Facebook page as well, as best as I could, to at least let our customers know that we were going to be opening and what days we would be open. Because there were a couple that we were able to come in, and then some I just couldn't get a pharmacist so we had to close." [A25] ; "Our PHN contact joined our Facebook group. So when we had collective or common messages to pass we used Facebook. Sometimes, we were asking questions of our PHN and the LDCC that we knew all the other pharmacies would benefit from the answer. So we asked the question publicly." [C232] ; "We did comment or post on their (LDCC) Facebook page, and also on a couple of local radio Facebook pages , but that seemed to get word out that we were one of very few pharmacies actually open." [L271] ; "…because we're a relatively small community (pharmacists), we have a Facebook group chat. There's someone from every pharmacy in it. In the end pharmacists were just messaging on there saying is anyone able to work this shift at this centre, or is anyone able to come help me at this time at this centre." [N212] ;*
40	Communication through Northern Qld Primary Healthcare Network	*"It was co-ordinated through originally the – the pharmacists were co-ordinating it and keeping a list of who was open. Everything like that. Then the PHN - the Primary Health Network took over and they started doing emails out of who was open. What their staffing levels were, what stock they had. Just to distribute it all around." [N212]*
41	Local Disaster Coordination Centre maintained communications	*"In the Local Disaster Co-ordination Centres they've got operations cells, - they've got health cells. And that's where we tap into – also your Primary Health Networks. Our PHN (Northern Queensland PHN) is tapped into that health cell of the Local Disaster Co-ordination Centre as well. So then that health cell has got the PHN and HHS all integrated." [C232]*
42	Lack of knowledge of which Government Organisation to contact in future disasters	*"Not specifically, no. I mean in that case if I was able to I would just Google the emergency services and contact like a hotline number I assume they have." [L271] ; "No I wouldn't actually." [N212] ; "I can't remember the name of it though." [A25]*
43	Call for Pharmacists to work in emergency evacuation centres	*"They (LDCC) actually put out a call for pharmacists to go to all the disaster centres. So a couple of my friends – I couldn't because we were still one of the pharmacies that were open. But there were pharmacists that couldn't get to their own pharmacies or even their houses had been flooded and they worked in the disaster centres sorting out people's medication. I never really knew that was a thing until it happened." [N212]*
44	Pharmacy provided medical supplies to LDCC	*"We did provide information to LDCC, and we also supplied them with hand sanitiser, bandages, basic first aid stuff like that. Hydralyte, things like that to them to provide to volunteers and people that were coming through seeking assistance." [Y52]*
45	Support from Professional Bodies	*"In preparation phase the Guild and the PSA are great. The Guild especially for us as business owners provide support for emergency management plans." [C232]*
