# Supplementary material for: An Exploration of the Impacts of the 2019 Floods in Townsville, Australia on Community Pharmacy Operations
Source: Prehosp Disaster Med. 2025 Aug 27;40(4):232–8. doi: 10.1017/S1049023X25101301 (PMC12809201; doi:10.1017/S1049023X25101301)
Supplement: Singleton et al. supplementary material 3 — Singleton et al. supplementary material [file S1049023X25101301sup003.pdf]

Methods

This qualitative research study utilised semi-structured interviews. The interview questions (Supplementary File 1) explored pharmacists' experiences of the flood event and included questions relating predominantly to the Response phase of the Prevention-Preparedness-Response-Recovery (PPRR) disaster management model.[6] These interview questions have been employed in two previously published studies.[7, 8] The reporting of this study follows the Consolidated Criteria For Reporting Qualitative Research (COREQ) Guidelines (Supplementary File 2).[9] Prior to data collection, ethical approval was obtained from the Queensland University of Technology Human Research Ethics Committee (Approval Number 1900000230).

Sampling Strategy and Participant Recruitment

To identify affected pharmacies, flood data were matched to the corresponding geographical locations. Fourteen pharmacies were identified as potentially flooded or cut off by floodwaters. Some of these pharmacies were independently owned and some belonged to three different larger pharmacy groups; pharmacists working for these groups were able to confirm affected pharmacies which eliminated four pharmacies. The remaining 10 pharmacies were then called with seven pharmacies' owners/managers agreeing to their staff being interviewed. Eligibility criteria for participants were that they had to be a registered pharmacist with the Australian Health Practitioner Agency (AHPRA) and have been working in Townsville between 29/1/2019 – 11/2/2019 in community pharmacies which were affected in some way by flooding. Pharmacists who were willing to participate then signed an Informed Consent document and an interview time was arranged. Participants were also asked if they knew of any other pharmacists who met the eligibility

criteria and might be willing to participate in the research. A total of six pharmacists were interviewed.

Data collection and analysis

KW and JS conducted the interviews over the phone. Both KW and JS are registered pharmacists with AHPRA but were not known to any of the interviewees. Only the interviewee and either KW or JS were present during the interviews. Each interview was digitally recorded and averaged 20 minutes with a range of 9-52 minutes. No repeat interviews were undertaken. To ensure confidentiality, prior to commencement of their interview each participant was assigned a unique ID code which was used in the audio recording. The audio files were then transcribed using *intelligent verbatim* by AL. The transcripts were not returned to participants for comment. Since participants' responses often addressed more than one question at a time, the interviews were analysed in their entirety rather than analysing each question's data separately. AL and JS analysed the data using reflexive thematic analysis.[9] Each participant's interview transcript was coded as a whole.

Several passes of the data were undertaken by AL and JS independently in the manual coding phase utilising an open coding technique[10] to derive codes. Whilst a predominantly deductive approach was taken mapping back to the P-P-R-R framework, a formal deductive approach such as grounded theory (as per Corbin and Strauss)[9] was not used as we were open to new emerging themes. AL and JS then compared codes. JS was able to provide professional insight into some of the participants' responses for clarity whilst AL not being a pharmacist reduced the risk of bias. Through discussion a single set of codes was derived and then condensed into a smaller number of categories (sub-themes)

and finally into key themes.[9, 10] EM and KW provided feedback on the allocation of codes to data and also on the coding tree with revisions made through team discussions.
